# Malaysian School Counselor’s Self-Efficacy: The Key Roles of Supervisor Support for Training, Mastery Experience, and Access to Training

**DOI:** 10.3389/fpsyg.2021.749225

**Published:** 2021-12-13

**Authors:** Pei Boon Ooi, Wan Marzuki Wan Jaafar, Glenda Crosling

**Affiliations:** ^1^Department of Medical Sciences, School of Medical and Life Sciences, Sunway University, Subang Jaya, Malaysia; ^2^Department of Counselor Education and Counseling Psychology, Faculty of Educational Studies, Universiti Putra Malaysia, Seri Kembangan, Malaysia; ^3^Centre for Higher Education Research, Sunway University, Subang Jaya, Malaysia

**Keywords:** access to training, counseling self-efficacy, job satisfaction, Malaysian school counselors, mastery experience, perceived supervisor support of training

## Abstract

The concept of self-efficacy has been widely studied and shown to contribute to individuals’ job satisfaction. For counselors, the concept measures their belief in their ability to conduct counseling sessions. However, it is an understudied area. As Bandura states, self-efficacy and its sources should be investigated and measured within its domain, which in this case is school counseling. This study examined the impact on school counselors’ self-efficacy and job satisfaction of the personal and environmental factors: (a) mastery experience, (b) social persuasion, (c) vicarious learning, (d) physiological and affective state, (e) the access to training, and (f) perceived supervisor support of training. The cross-sectional study involved 541 Malaysian secondary school counselors nationwide via a random sampling-distributed questionnaire. Results which were analyzed using PLS-SEM, with importance-performance functionality embedded in it, indicated that mastery experience, access to training, and perceived supervisor support of training explained 45.6% variance in counseling self-efficacy and together with counseling self-efficacy, contributed 13.2% variance in job satisfaction among the school counselors. The importance-performance map analysis revealed supervisor support of training as of greatest importance in shaping counseling self-efficacy. Counseling self-efficacy partially mediated the relationship between mastery experience, access to training, supervisor support toward training, and job satisfaction Arising from this finding is a proposed theoretical framework in which efficacy information (i.e., mastery experience), environmental determinants (i.e., access to training and supervisor support of training) and cognitive determinant (i.e., counseling self-efficacy) corresponded together congruently and lead to higher job satisfaction. Suggestions are also made for training providers, content developers, and policymakers to include these factors in professional development training and continuous education, to sustain the wellbeing of school counselors.

## Introduction

The focus of this study is the examination of Malaysian school counselors’ (SC) job satisfaction in relation to counselors’ sense of self-efficacy. Increasingly integral to education systems internationally including in Malaysia (Ooi et al., 2018), SCs address students’ guidance and counseling needs. While the concept of self-efficacy has been studied (Lin et al., 2020) and generally seen as contributing to educators’ job satisfaction (Burić and Moe, 2020; Demir, 2020), counseling self-efficacy, CSE (i.e., how well a counselor believes in their ability to conduct counseling sessions) is understudied in the field. Additionally, CSE predicted counselors’ professional commitment to the profession, students, and organization (Akinlolu and Chukwudi, 2019).

Responsible for clinical counseling services such as emotional counseling and group intervention, SCs cover the five domains of academic, career, psychosocial and mental health wellbeing, stakeholders’ management, and student discipline (Al Haikal et al., 2020). Self-efficacy supports SCs’ job satisfaction via their ability to perform their jobs with interest and motivation, providing increasingly effective services. As a domain-specific notion of the general self-efficacy concept, CSE refers to belief or judgment in ability and competency to effectively perform counseling tasks/activities in the near future (Larson and Daniels, 1998; Watson, 2012). A domain-specific self-efficacy approach, rather than general, predicts effective and quality clinical counseling practices and better client outcomes and satisfaction (Schiele, 2013). For Bandura (1977), self-efficacy management should be domain-specific.

Job satisfaction for SCs and self-efficacy are linked, as evidenced in the definition of SCs’ self-efficacy: “… belief or judgments about his or her expectation to effectively counsel a client” (Larson and Daniels, 1998, p. 180). While in the past study, a significant positive relationship exists between SCs’ general self-efficacy and job satisfaction (Aliyev and Tunc, 2015), studies of counselors’ counseling self-efficacy and job satisfaction are not reported.

For individuals constructed their self-efficacious information from the four sources: enactive mastery experience, vicarious experience, social persuasion, and physiological and affective states. Depending on circumstances, one or more than one source can influence self-efficacy more highly than the others (Bandura, 2012b). Apart from mastery experience, no consistent empirical evidence supports the sequence of the other three sources, and contextualization seen in the study in an integrated context is needed. This study adopts the Malaysian context-validated Sources of Counseling Self-Efficacy—Malaysia Scale (SCSE-M) which reports good internal consistency (Ooi et al., 2020).

The self-efficacy sources function concurrently with the cognitive process and integrated information that determines an individual’s self-efficacy beliefs (Britner and Pajares, 2006), subsequent environmental interaction, and motivational factors (Bandura, 1991). These underpin learning and achievement-related behavior, including successful counseling sessions and task performance. The COVID-19 pandemic has highlighted the importance of SCs’ self-belief, including in Malaysia (Yusoff et al., 2020), in managing changing school dynamics of lockdown and home-based learning, remotely supporting stressed students mentally and socially. Self-efficacy beliefs underpin counselors’ confidence and motivation to address challenges and experience job satisfaction. Furthermore, in Malaysia, as SCs’ role has evolved only over the past half-century (Abdul Rahman et al., 2013; Low et al., 2013), a knowledge gap exists in SCs’ self-belief and self-efficacy. This amplifies the need for this study.

In this study, we test a model of CSE sources and examine SCs’ self-efficacy as potentially shaping job satisfaction. We explore the relationships of the self-efficacy sources and job satisfaction, with counseling self-efficacy as a mediator. We urge that personal and environmental factors contribute to CSE and thus predict job satisfaction. The study in an area of very limited research contributes to a gap in understandings in the field. Its significance is underscored in Malaysia’s relatively new SC setting which increasingly in current times, impacts school communities’ wellbeing. With the vital role of school communities in societies including in Malaysia in preparing young citizens, enhanced school operations as impacted by counseling roles justify the value of this study.

## Theoretical Background

This study of the impact of self-efficacy on job satisfaction for Malaysian SCs is framed by the Social Cognitive Theory (SCT), where the self-extensively motivates and regulates behavior (Bandura, 2012a; Schunk, 2017; Schunk and DiBenedetto, 2020). In SCT, behavior regulation for work actions and approaches shifts to the individual (Bandura, 1977; Chang and Edwards, 2015), as distinct from earlier understanding that behavior was shaped by action reinforcement and immediate consequences, not involving the person. In this study, SCT is used to address counselors’ self-efficacy because counselors are largely responsible for their schoolwork actions and behavior. Their work contrasts with other forms of work that are closely supervised by other staff and so involve a higher level of reinforcement and immediate consequences. Furthermore, self-regulation as key in SCT is significant in learning as it drives learner interests, tenacity, and motivation (Bandura and Walters, 1977; Schunk and DiBenedetto, 2020) and as such can be seen as relevant to job satisfaction. As self-efficacy beliefs concern individuals’ perceived capabilities to produce results with designated types of performance, the focus is different from conceptions of personal competence, which are core in other theories. Self-efficacy judgments are both more task- and situation-specific (contextual), as is the situation with counselors and their school actions. This again emphasizes the key role and high relevance of SCT in this study. As such, individuals use these judgments for a goal.

Self-efficacy beliefs are powerful in shaping achievements and influencing aspirations. Bandura (1986) stated that knowing how and having the task skills do not guarantee performance accomplishment. Furthermore, humans do not always strive for optimum performance, although they have “know-how” to do so, because of self-efficacy thought, which may impact their self-regulation in the implementation of their work tasks and activities. Self-efficacy beliefs, therefore, mediate outcomes between an individual’s action and their know-how (Iroegbu, 2015). In sum, self-efficacy refers to the individual’s “…judgment on their capability to organize and execute the courses of action required to attain designated types of performance” (Bandura, 1986, p. 391).

This study, which links SCT theory with counselors’ self-efficacy, is undertaken where other studies in the field are scarce and, importantly, are not contextualized to the *sources* of counseling self-efficacy. An in-depth study of self-efficacy in the counseling context such as this study is thus warranted to support SCs’ optimum operation in Malaysian public schools. While CSE studies have been widely examined among counselor candidates (Bakioglu and Türküm, 2020), Master’s-level counseling interns and doctoral counseling students (Akinlolu and Chukwudi, 2019), and counseling teachers (Harun, 2015), studies of Malaysian secondary SCs are very limited.

Additionally, most research has posited non-counseling-related self-efficacy or general self-efficacy as a mediator. For example, the use of teacher self-efficacy as a mediator between emotional job demands of teaching and wellbeing among teachers (Huang et al., 2019) or general self-efficacy mediates the relationship between CSE and positive-negative emotions among groups of psychological counselor candidates (Ümmet, 2017). A recent study by Ellis (2019) reported on the mediating role of counselors’ self-efficacy and career sustaining behaviors and burnout but not job satisfaction and the result also indicated that counselors’ self-efficacy did not act as the mediator to the relationship.

Thus, a limited number of studies of CSE as the mediator supports the relevance of this study, especially in Malaysia where SCs’ evolving role has faced challenges in managing current situations (Ooi et al., 2018). Evidence of the evolution of the role is where pre-COVID-19, was solely a guidance and counseling service provider, but during COVID-19, SCs support students in the context of virtual learning, mental health issues, and disruption (Mahomed et al., 2019; Ku Johari et al., 2020; Tan, 2021).

### Self-Efficacy and Sources

Studies of self-efficacy’s impact on behavior and performance quality, that is, its measurement, show the relevance of specific self-efficacy domains, rather than the generalized concept. As Bandura (1993, p. 118) states, the individual ability is not ‘a fixed attribute’, and individuals construct their sense of self-efficacy (their efficacious information) from four primary sources: mastery experience (personal experience of success); vicarious experience (observing others as successful role models); social or verbal persuasion (others’ comments that the individual is capable of mastering the activity), and physiological and affective states (positive emotions boosting self-confidence inability to succeed) (Bandura, 1997).

Bandura (1997) explains that different results arise from the relationship between these four sources and self-efficacy, depending on contextual factors; in an individual’s work-related performance, of the four sources, mastery experience is the most influential (Zelenak, 2015; Capa-Aydin et al., 2018) in enhancing self-efficacy and thus performance (Wilson et al., 2020). Mastery experience is based on an individual’s authentic experience and performance (Phan and Ngu, 2016) where success strengthens self-efficacy and failures, especially early in the experience, weaken self-efficacy. Additionally, Hu et al.’s (2015) finding reported that counselors’ mastery experience predicts their client-specific self-efficacy: it significantly correlated with the level of counselor-client goals and delivered better therapeutic results. Thus, we hypothesized:

Hypothesis H_1_: There is a significant positive relationship between mastery experience and counseling self-efficacy among school counselors.

Social persuasion or verbal persuasion alone is unable to sustain an individual’s self-efficacy (Bandura, 1986). However, verbal persuasion could bolster self-efficacy through compliments, praise, or confirmation from a significant person when the individual faces challenges or self-doubt. Overall, persuasory efficacy information may boost the individual’s sense of efficacy (Capa-Aydin et al., 2018) and enhance an individual’s occupational expectations (Talsma et al., 2018). Social persuasion takes the role of a booster to strengthen one’s belief, particularly when it was done within a reasonable range of situations (i.e., individual perceived moderately in the persuasory efficacy appraisals) (Bandura, 1997; Conner and Norman, 2015). When faced with difficult situations, but being encouraged or given the booster, the sense of efficacy, therefore, was easy to sustain and resulted in a greater effort to master the assigned responsibilities and tasks. Thus, we hypothesized:

Hypothesis H_2_: There is a significant positive relationship between social persuasion and counseling self-efficacy among school counselors.

Individuals’ self-efficacy is also enhanced through vicarious learning—observing another person similar to themselves achieving successful task performance—which helps an individual make wise decisions based on the costs and consequences involved (Morris et al., 2017). One’s self-efficacy is raised further when others who have similar skills and competencies performed a task successfully (Bandura, 1986). However, if a person of similar character and competency fails, an individual would be cautious in repeating or imitating that person’s behavior. The greater the similarity, the greater the persuasive power of the vicarious model to enhance self-efficacy (Gundel and Piro, 2021). Thus, we hypothesized:

Hypothesis H_3_: There is a significant positive relationship between vicarious learning and counseling self-efficacy among school counselors.

Physiological and affective states are somatic information, referred to as the emotional state experienced such as anxiety associated with task performing and thus undermined the sense of self-efficacy (Lewandowski, 2019; Peura et al., 2021). Fatigue, windedness, aches, and pains were associated with inefficacy among school teachers (O’Neill and Stephenson, 2012; Morris et al., 2017). However, these findings were merely on teachers and not among SCs. Underpinned by these studies among teachers, we hypothesized:

Hypothesis H_4_: There is a significant negative relationship between physiological and affective state and counseling self-efficacy among school counselors.

### Access to Training, Perceived Supervisor Support of Training and Counseling Self-Efficacy

Professional learning opportunities contribute positively to an individual’s self-efficacy. A study of 180 Greek school career counselors found counselors showed higher confidence, felt empowered after having adequate training access and opportunities, and as a result, it increased their professional efficacy and competencies (Kounenou et al., 2010, 2018). Additionally, programs such as counseling preparation programs or continuous professional training attended by counselors, too, increased their self-efficacy (Malkoç and Sünbül, 2020; Perry et al., 2020). Thus, we hypothesized:

Hypothesis H_5_: There is a significant positive relationship between access to training and counseling self-efficacy among school counselors.

School counselors’ counseling self-efficacy in their capabilities to perform counseling tasks relate to the nature of the counselor-supervisor working relationship -i.e., positive relationships promote higher self-belief and produce better clients’ therapeutic outcomes (Morrison and Lent, 2018). Additionally, an environmental climate including aspects such as university facilities, social support, supervision, and environmental factors was found to correlate highly and significantly with counseling self-efficacy (CSE) (Bagheri et al., 2012). Access to training programs may impact the supervisory working alliance and psychotherapy process and outcome (Keum and Wang, 2021). For SCs, their supervisor’s role in providing equal resource access such as training is imperative in maintaining work effort, especially when it contributes to the therapeutic relationship, client satisfaction, and treatment outcomes (Keum and Wang, 2021). The greater the support from their supervisor, the greater the sense of efficacy at work. Thus, we hypothesized:

Hypothesis H_6_: There is a significant positive relationship between perceived supervisor support of training and counseling self-efficacy among school counselors.

### Counseling Self-Efficacy and Job Satisfaction

The research on the relationship between CSE and job satisfaction is limited. The closest theme seen in the literature is general self-efficacy and job satisfaction, which has been studied widely. While self-efficacy does not measure competency, a higher self-efficacy level corresponds with higher performance, as self-efficacy increases motivation for task completion because of a greater accomplishment rate (Bandura, 1986; Honicke and Broadbent, 2016).

Aliyev and Tunc (2015) reported a significant and positive relationship between self-efficacy and job satisfaction among school psychological counselors, and the sense of self-efficacy was closely related to how SCs self-regulate and self-evaluate their performance and behavior in performing counseling related tasks. School counselors who feel efficacious of their competency presented themselves as optimistic and believe in their capabilities to conduct counseling tasks, duties, and responsibilities, and thus reported higher job satisfaction. School counselors who feel efficacious about their competency presented as optimistic and believed in their capabilities for counseling tasks, duties, and responsibilities and sense of job satisfaction were easier to be accomplished through the process. Thus, we hypothesized:

Hypothesis H_7_: There is a significant positive relationship between counseling self-efficacy and job satisfaction among school counselors in Malaysia.

**FIGURE 1:**
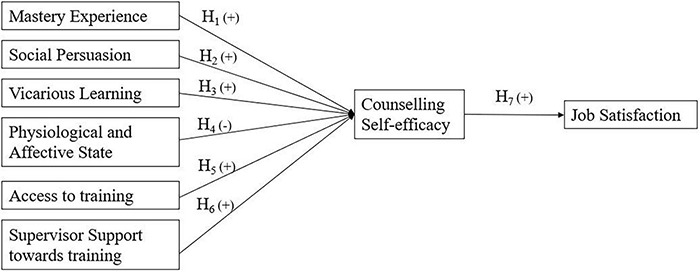
Proposed research model.

Considering all the above-mentioned hypotheses, we propose the below research model (Figure 1).

## Materials and Methods

### Sample and Procedure

This cross-sectional study adopted an anonymous self-report dual language (English and Bahasa Malaysia) questionnaire method, administered nationwide over 6 months to 1,000 SCs. The English version was translated into Bahasa Malaysia through a series of validation processes, firstly by two bilingual professional independent certified translators for forwarding translation (English to Bahasa Malaysia). The authors addressed the forward translation’s discrepancies; the revised version’s backward translation was then undertaken by two different professional independent certified translators (Bahasa Malaysia to English). After review by three experts (a psychologist with 25 years of clinical experience, a counselor with 8 years experience, and an associate professor in counseling with 11 years experience), the final version generated showed satisfactory face and semantic, criteria and conceptual equivalence in English and Bahasa Malaysia so participants could answer in either language.

Aligned with Hair et al.’s (2010) suggestion of a minimum number of 500, this study’s minimum sample of 500 complies with both research design and study objectives. The list of secondary schools in Malaysia available on the Malaysian Ministry of Education’s website was keyed into the online randomization tool^1^ and the first 600 schools were sampled, taking into consideration the dropout rate of 20%.

With the school list generated, the researcher has the questionnaire mailed out. Each set of envelopes was equipped with return stamps, a return envelope, a standard participant information sheet containing the objectives of the study, supporting letters from both the Ministry of Education, Malaysia, and the state education department, respectively, as well as the ethics approval letter. Follow-up calls were conducted 3 weeks after the questionnaire was mailed to the schools’ addresses of each state. The 3 weeks duration has taken into consideration five administrative steps before a response could be mailed back to the researcher, (a) the mail must first arrive at the administrative office of the school, (b) approval granted by the school principal, (c) disseminating to any school counselor randomly (d) answering the questionnaire, and (e) returned mail time. The random sampling-distributed method was used as each school has more than one (1) counselor and the questionnaire could be distributed by the school principal to any of the counselors. Any school counselor who has been working as a counselor in the school system is eligible to participant in the study.

Approval and ethics clearance was obtained before data collection from the Ministry of Education Malaysia and respective educational state departments and schools. A test of non-response bias was conducted by comparing respondents who returned the questionnaires in the first 3 months and those who replied after the 3rd month. The differences were not significant and thus we concluded that non-response bias is not a concern for this study.

The total number of questionnaires returned was 560 (89.6%) with 541 responses being useful for analysis and 19 returned questionnaires with missing data rejected (more than 15% of the entire questionnaire) (Isaac et al., 2017). The final sample size was considered adequate.

A total of 541 school counselors (77.8% women) based in Malaysian secondary schools participated in this study, with a mean age of 39.34 (*SD* = 8.89). Most participants, i.e., 348 (64.3%), were Malay, 140 (25.9%) Chinese, 40 (7.4%) Indian, and 13 (2.4%) belonging to ‘others’ (the indigenous groups in Malaysia), comprised of five Iban, three Bidayuh, two each for Dusun and Murut, and one Tatau. Most participants (422; 78%) hold an undergraduate degree. Among the total number of 111 with a postgraduate degree, 104 (19.22%) have a master’s, and seven (1.29%) have a doctorate. A small percentage (1.49%) possessed other qualifications such as a diploma degree. All participants currently have at least 3 years of experience and provide counseling services at schools.

Among the variables, social persuasion reported the higher mean score (*M* = 5.35, *SD* = 0.425), and physiological and affective state the lower mean score (*M* = 1.64, *SD* = 0.555) (Table 1).

**TABLE 1:** Means, standard deviations, correlation of study variables.

Variable	M	SD	Min	Max	1	2	3	4	5	6	7	8	9
Mastery experience	4.88	0.704	1.00	6.00	**1**								
Social persuasion	5.35	0.425	3.00	6.00	0.126*	**1**							
Vicarious learning	5.15	0.522	3.57	6.00	0.167**	0.526**	**1**						
Physiological and affective state	1.64	0.555	1.00	3.66	0.119*	0.053	–0.014	**1**					
Access to training	4.63	0.537	3.00	5.00	0.206**	0.060	0.057	–0.050	**1**				
Supervisor support of training	4.70	0.508	3.00	5.00	0.136*	0.093*	0.036	0.024	0.579**	**1**			
Counseling self-efficacy	4.49	0.583	2.67	6.00	0.414**	0.150*	0.113*	–0.029	0.563**	0.564**	**1**		
Job satisfaction	3.98	0.538	1.00	5.00	0.223**	0.031	0.062	–0.081	0.188*	0.202**	0.440**	**1**	

N = 541; Min, minimum; Max, maximum; M, Mean; SD, Standard deviation; *p < 0.05, **p < 0.001. Bold indicates the correlation between the same variable.

### Measures

The Sources of Counseling Self-Efficacy Scale (SCSE-M) was adopted from the “Source of Middle School Mathematics Self-Efficacy Scale” by Usher and Pajares (2009) with 25 items on a scale ranging from 1 (definitely false) to 6 (definitely true) and item 14 being reverse scored. For the present study, the Cronbach’s Alpha of the Scale for each domain, was 0.868 (Mastery Experience), 0.878 (Social Persuasion), 0.933 (Vicarious learning), and 0.921 (Physiological and affective state). Each scale’s total mean score was used for analysis. The psychometric properties of SCSE-M and the adaptation and modification were deemed appropriate to allow the sources of self-efficacy to function and measure the construct in the domain-specific counseling profession (Ooi et al., 2020). Examples are “I do well on even the most difficult counseling sessions” and “Other colleagues have told me that I’m good at doing counseling.”

The access to training and perceived supervisor support of training components, measured by Bulut and Culha (2010) nine items and five Likert scales (1 strongly disagree, to 5 strongly agree) and no reverse scores required. The Cronbach’s Alpha value of access to training and perceived support of training for the present study was 0.770 and 0.962, respectively. A mean score was used. Examples are “This organization provides access to training” and “My supervisor enthusiastically supports my participation in training programs.”

The Counseling Self-Estimate Inventory (COSE) measured counseling self-efficacy (Larson et al., 1992) with a 6 point Likert scale on how they feel they will behave as a counselor in a counseling situation for each item. The scale ranged from 1 (strongly disagree) to 6 (strongly agree). Nineteen (19) items (item 2, 6, 7, 9, 16, 18, 19, 21, 22, 23, 24, 26, 27, 28, 31, 33, 35, 36, and 37) required reverse scored. For the present study, Cronbach’s alpha coefficients reported for the COSE total score was α = 0.93 and the five subdomains were 0.88 for micro-skill, 0.87 for processing, 0.8 for dealing with difficult client behavior, 0.80, 0.78 for cultural competence, and 0.62 for awareness of the value (Larson et al., 1992). Mean score was used and examples are “I feel competent regarding my abilities to deal with crises that may arise during the counseling sessions, e. g., suicide, alcoholism, abuse, etc.” and “I am confident that I will know when to use open or closed-ended probes and that these probes will reflect the concerns of the client and not be trivial.”

The Minnesota Satisfaction Questionnaire (MSQ), short-form, developed by Weiss et al. (1967) measured the sense of job satisfaction by 20 items and runs on a five-point Likert scale with responses from 1 (very dissatisfied) to 5 (very satisfied). The present study’s Cronbach’s alpha coefficients were 0.894 for intrinsic satisfaction and 0.834 for extrinsic satisfaction. A mean score was used with a percentage score of 75 or higher representing a high degree of satisfaction. Examples are “My pay and the amount of work I do” and “the feeling of accomplishment I get from the job.”

## Data Analysis and Results

### Data Analysis

Partial least squares structural equation modeling (PLS-SEM) was used, with the SmartPLS version 3 as the statistical tool to examine the measurement and structural model (Henseler et al., 2015). The PLS-SEM has emerged as a statistical tool for psychology and social sciences in examining both measurement and structural models in survey studies, which usually are not normally distributed (Chin et al., 2003; Hair et al., 2017; Ramayah et al., 2018).

As our data used solely a single source, we first tested the issue of Common Method Bias according to Kock and Lynn (2012) by testing the full collinearity. All the variables will be regressed against a common variable and if the VIF ≤ 3.3, there is no bias from the single-source data. Our results yielded a VIF of less than 3.3, thus single-source bias is not a serious issue with our data (Kock and Lynn, 2012). Additionally, the construct measured use different Likert-type response scales, respectively, (5-point, 6-point, and 7-point) to control common method bias effects (Tehseen et al., 2017).

We then examined the measurement model (i.e., to test the instrument’s validity and reliability) and structural model (i.e., to test the hypothesis developed) to finalize the outcome with the bootstrapping method of 5,000 resamples (Hair et al., 2017).

### Measurement Model

The assessment of the measurement model examined the convergent validity and the discriminant validity. Convergent validity is assessed via factor loading, average variance extracted (AVE), and composite reliability (CR) (Hair et al., 2017). A total of 22 items were dropped due to low factor loading (i.e., 7 items for counseling process; 4 items each for micro-skills; 3 items each for both intrinsic and extrinsic satisfaction; 2 items for dealing with difficult clients; 1 item each for value, cultural competency, and perceived supervisor support toward training). Since the study had two second-order constructs of (1) Counseling Self-Estimate Inventory and (2) Minnesota Job Satisfaction, the validity and reliability of the second order was also examined (Table 2). All criteria were satisfactory as the item loadings were higher than 0.6, the AVE was higher than 0.5, and the values of CR were above 0.7. Thus, the convergent validity for scale measurement is fulfilled for both first and second-order constructs.

**TABLE 2:** Convergent validity.

First order constructs	Second order construct	Item	Loading	CR	AVE
Mastery experience (MA)		MA1	0.723	0.905	0.614
		MA2	0.765		
		MA3	0.839		
		MA4	0.862		
		MA5	0.793		
		MA6	0.706		
Social persuasion (SP)		SP1	0.768	0.908	0.622
		SP2	0.773		
		SP3	0.745		
		SP4	0.776		
		SP5	0.843		
		SP6	0.825		
Vicarious learning (VL)		VL1	0.863	0.944	0.708
		VL2	0.848		
		VL3	0.844		
		VL4	0.865		
		VL5	0.825		
		VL6	0.809		
		VL7	0.839		
Physiological and affective states (PH)		PH1	0.834	0.930	0.689
		PH2	0.883		
		PH3	0.761		
		PH4	0.756		
		PH5	0.859		
		PH6	0.878		
Access to training (ACT)		ACT1	0.828	0.869	0.689
		ACT2	0.869		
		ACT3	0.793		
Supervisor support of training (SUP)		SUP1	0.990	0.969	0.841
		SUP2	0.978		
		SUP3	0.971		
		SUP4	0.981		
		SUP5	0.926		
Dealing with difficult client (CLT)		CLT1	0.906	0.902	0.651
		CLT3	0.680		
		CLT4	0.780		
		CLT6	0.739		
		CLT7	0.904		
Microskill (MC)		MC3	0.687	0.918	0.584
		MC6	0.731		
		MC7	0.850		
		MC8	0.834		
		MC9	0.818		
		MC10	0.803		
		MC11	0.681		
		MC12	0.686		
Cultural competency (CUL)		CUL1	0.843	0.886	0.721
		CUL3	0.831		
		CUL4	0.873		
Value (VAL)		VAL1	0.900	0.933	0.824
		VAL3	0.855		
		VAL4	0.965		
Counseling processing (CP)		CP8	0.858	0.833	0.716
		CP9	0.841		
		CP10	0.840		
	Counseling self-estimate Inventory	CLT	0.949	0.922	0.708
		MC	0.944		
		CUL	0.880		
		VAL	0.757		
		CP	0.634		
Intrinsic satisfaction (INT)		M3	0.722	0.923	0.547
		M4	0.701		
		M7	0.732		
		M8	0.739		
		M9	0.755		
		M10	0.704		
		M11	0.745		
		M15	0.795		
		M16	0.808		
		M20	0.683		
Extrinsic satisfaction (EXT)		M5	0.705	0.851	0.656
		M6	0.692		
		M12	0.827		
		M13	0.825		
		M14	0.776		
	Minnesota Job Satisfaction	INT	0.646	0.912	0.839
		EXT	0.639		

*AVE, Average variance extracted; CR, Composite reliability.*

As Henseler et al. (2015) suggest, heterotrait-monotrait (HTMT) ratio of correlations based on the multitrait-multimethod matrix was used to assess discriminant validity (Henseler et al., 2015). When the confidence interval contains the value 1 (i.e., H_0_ holds)- a lack of discriminant validity is reported. In the present study, all the values are below the threshold level, HTMT_0.90_ (Gold et al., 2001), and also the HTMT Inference shows that the confidence interval did not show a value of 1 on any of the constructs, indicating that discriminant validity has been determined (Table 3).

**TABLE 3:** Heterotrait-monotrait (HTMT) ratio.

	1	2	3	4	5	6	7	8
Access to training								
Counseling self-efficacy	0.569							
Job satisfaction	0.182	0.406						
Mastery experience	0.214	0.44	0.233					
Physiological and affective state	0.062	0.051	0.085	0.134				
Social persuasion	0.077	0.171	0.048	0.135	0.08			
Supervisor support of training	0.59	0.568	0.213	0.137	0.023	0.09		
Vicarious learning	0.067	0.121	0.044	0.168	0.04	0.534	0.033	

### Structural Model

Following Hair et al. (2017), R^2^, standard beta, *t*-values *via* a bootstrapping procedure with a resample of 5,000 was conducted and reported. The effect sizes (f^2^) and the predictive relevance (Q^2^) assessed the structural model (Table 4). Statistical results revealed that 5 of the 7 hypotheses were supported and 2 not supported. Master experience (β = 0.299, *p* < 0.01), social persuasion (β = 0.07, *p* < 0.01), access to training (β = 0.235, *p* < 0.01), and supervisor supports toward training (β = 0.381, *p* < 0.01) have positive relationship with counseling self-efficacy. Additionally, counseling self-efficacy reported a positive relationship with job satisfaction (β = 0.363, *p* < 0.01). Therefore, H_1_, H_2,_ H_5_, H_6_, and H_7_ are supported whereas H_3_ and H_4_ are not supported.

**TABLE 4:** Structural model.

Hypothesis	Path relationship	Std. beta	Std Error	*t*-value	Decision	Confidence Interval (BC)		VIF	*R* ^2^	*f* ^2^	Effect size	*Q* ^2^
						LL	UL					
Direct relationship												
H_1_	Mastery experience—> counseling self-efficacy	0.299	0.04	7.552**	Supported	0.225	0.383	1.072	0.456	0.154	Medium	0.311
H_2_	Social persuasion—> counseling self-efficacy	0.070	0.038	1.769**	supported	0.005	0.148	1.319		0.007	No effect	
H_3_	Vicarious learning—> counseling self-efficacy	0.012	0.034	0.369	Not supported	–0.076	0.065	1.328		0.000	No effect	
H_4_	Physiological and affective state—> counseling self-efficacy	–0.066	0.049	1.351	Not supported	–0.13	0.05	1.018		0.008	No effect	
H_5_	Access to training_—> counseling self-efficacy	0.235	0.046	5.067**	Supported	0.146	0.326	1.417		0.073	Small	
H_6_	Supervisor support_—> counseling self-efficacy	0.381	0.042	9.14**	Supported	0.299	0.462	1.396		0.152	Medium	
H_7_	Counseling self-efficacy—> job satisfaction	0.363	0.04	9.166**	Supported	0.288	0.441	1.000	.132	0.195	Medium	0.106
**Mediation relationships**												
	Mastery experience—> counseling self-efficacy—> job satisfaction	0.109	0.022	4.898**	Supported	0.069	0.158	Partial mediation				
	Social persuasion—> counseling self-efficacy—> job satisfaction	0.025	0.015	1.691	Not supported	–0.003	0.055	No mediation				
	Vicarious learning—> counseling self-efficacy—> job Satisfaction	0.005	0.014	0.332	Not supported	–0.022	0.032	No mediation				
	Physiological & affective state—> counseling self-efficacy—> Job satisfaction	–0.024	0.018	1.351	Not supported	–0.049	0.018	No mediation				
	Access to training—> counseling self-efficacy—> job satisfaction	0.086	0.019	4.458**	Supported	0.047	0.129	Partial mediation				
	Supervisor support—> counseling self-efficacy—> job satisfaction	0.139	0.022	6.356**	Supported	0.098	0.18	Partial mediation				

^**^p < 0.05; ^**^p < 0.01 This study also shows that counseling self-efficacy plays a significant role in partially mediating the relationship between mastery experience (β = 0.109, p < 0.01), access to training (β = 0.086, p < 0.01), supervisor support toward training (β = 0.139, p < 0.01) and job satisfaction (Table 4). No mediation effect was found for others (i.e., social persuasion, vicarious learning, and physiological affective state).

The *R*^2^-value is 0.456 for counseling self-efficacy and 0.132 for job satisfaction, respectively. Changes in *R*^2^-value were examined to determine effect size (*f*^2^), with 0.02.15, and 0.35, respectively, representing small, medium, and large effects (Cohen, 1988; Hair et al., 2017). Thus, the present study’s *f*^2^ effect sizes were only acceptable for four of the hypotheses -i.e., both mastery experience (*f*^2^ = 0.154), supervisor support of training (*f*^2^ = 0.152), have medium effects on counseling self-efficacy while access to training reported small effects on counseling self-efficacy (*f*^2^ = 0.073). Nonetheless, counseling self-efficacy reported medium effects on job satisfaction (*f*^2^ = 0.195) (Table 4).

**FIGURE 2:**
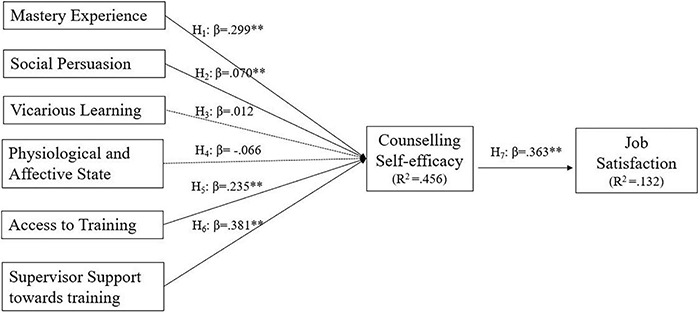
Final model result.

Following Henseler et al. (2009), the blindfolding procedure evaluated the proposed model’s predictive validity (Q^2^). This study reported *Q*^2^-values of 0.311 for counseling self-efficacy and 0.106 for job satisfaction. All Q^2^ reported suggested the model has sufficient predictive relevance since the value is more than 0 (Hair et al., 2017). According to Hair et al. (2017) the value of 0.02, 0.15, and 0.35 showed that an exogenous construct has a small, medium, or large predictive relevance on a certain endogenous construct (Hair et al., 2014, 2017). The result of the final research model is presented in Figure 2.

**FIGURE 3:**
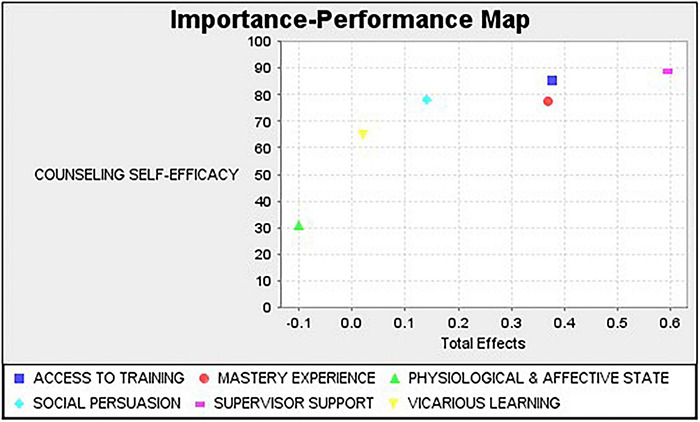
IPMA map of counseling self-efficacy.

To evaluate each construct’s actual performance in predicting counseling self-efficacy, the IPMA of counseling self-efficacy was conducted, finding that the construct physiological and affective state has had little to no context of shaping the counseling self-efficacy, being of low importance and performance (Figure 3).

The model’s most influential construct is supervisor support of training with the highest performance in predicting effect toward counseling self-efficacy, followed by access to training and mastery experience (Table 5).

**TABLE 5:** IPMA Result of counseling self-efficacy.

	Importance (Total effect)	Performances (Index values)
Access to training	0.376	85.221
Mastery experience	0.369	77.651
Physiological and affective state	–0.101	30.831
Social persuasion	0.14	78.274
Supervisor support	0.595	88.863
Vicarious learning	0.021	64.92

### Model Robustness

To test for model robustness, we first do the models comparison (e.g., proposed hypothesized model and alternative simpler model). Sarstedt et al. (2020) urged the researchers to perform model robustness checks in PLS-SEM to ensure that the proposed model does not suffer from problems of endogeneity (Rojo et al., 2016, 2018, 2020). The check includes the assessment of non-linear effects, endogeneity, and unobserved heterogeneity assessment.

### Models Comparison

According to Hair et al. (2019) and Sharma et al. (2021), the researcher can use Bayesian Information Criterion (BIC) (Schwarz, 1978) and Geweke-Meese Criterion (GM) (Geweke and Meese, 1981) to perform model comparison in PLS-SEM. In addition, PLS Predict in SmartPLS can also be used to generate root mean square error (RMSE) and mean absolute deviation (MAD) of the model (Shmueli et al., 2016). Sharma et al. (2021) stated that both criteria are suitable for model comparison when the researcher could opt for a holdout set with a large enough sample size. Therefore, we conducted the above-mentioned analyses to identify the best model by comparing the proposed model with an alternative model. The alternative model is a simpler model, in which the environmental variables (e.g., access to training and supervisor support) with a direct effect on job satisfaction without the mediating effect of counseling self-efficacy, while other variables remained constant.

**FIGURE 4:**
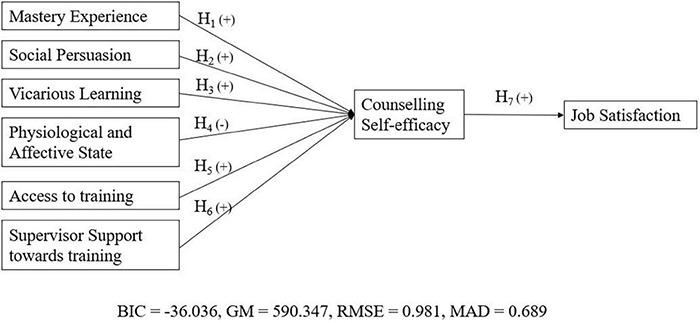
Proposed model.

**FIGURE 5:**
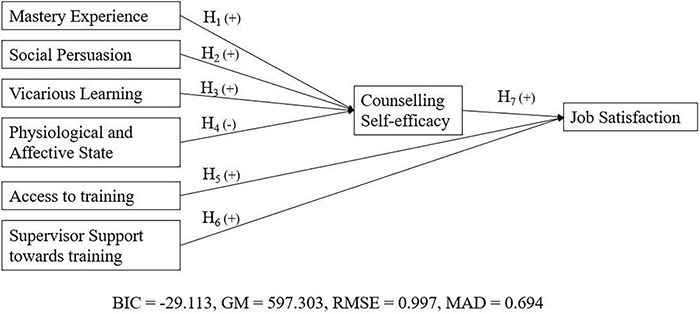
Alternative model.

The study of Hair et al. (2019) stated that the researcher should select a model which had a lower value of BIC and GM. In addition, Shmueli et al. (2019) stated that lower values of RMSE and MAD indicate higher predictive power of a model. Therefore, our results showed that the proposed model (Figure 4) with BIC = –36.036, GM = 590.347, RMSE = 0.981, MAD = 0.689 outperformed the alternative model (Figure 5) with BIC = –29.113, GM = 597.303, RMSE = 0.997, MAD = 0.694. Thus, we concluded that the proposed model provided a better explanation.

### Assessment of the Non-linear Effect

According to Svensson et al. (2018), researchers could conduct the Regression Equation Specification Error Test (RESET) (Ramsey, 1969) and calculate the interaction term of the quadratic effect of the structural model to measure the non-linear effect. With RESET, we found that the partial regression of counseling self-efficacy on mastery experience, social persuasion, vicarious learning, physiological and affective state, access to training, and supervisor support [*F*(2, 532) = 19.346, *p* = 0] is subject to non-linearity, but the partial regression of job satisfaction on counseling self-efficacy, mastery experience, social persuasion, vicarious learning, physiological and affective state, access to training, and supervisor support [*F*(1, 532) = 0.605, *p* = 0.437] do not have non-linearity effect (Table 6).

**TABLE 6:** Assessment of the non-linear effect.

Non-linear relationship	Coefficient	f^2^	*P*-values	Ramsey’s RESET
MA*MA – > CSE	0.042	0.010	0.216	***F* (2, 532) = 19.346, *p* = 0.000** *F* (1, 532) = 0.605, *p* = 0.437
SP*SP – > CSE	**0.035**	**0.007**	**0.019**	
VL*VL – > CSE	0.022	0.022	0.318	
PH*PH – > CSE	0.041	0.005	0.137	
ACT*ACT – > CSE	0.002	0.000	0.962	
SUP*SUP – > CSE	0.058	0.004	0.409	
CSE*CSE – > JS	–0.040	0.003	0.340	
MA*MA – > JS	–0.039	0.005	0.389	
SP*SP – > JS	0.019	0.001	0.499	
VL*VL – > JS	–0.033	0.002	0.250	
PH*PH – > JS	–0.030	0.002	0.421	
ACT*ACT – > JS	–0.018	0.000	0.645	
SUP*SUP – > JS	0.037	0.001	0.427	

MA, mastery experience; SP, social persuasion; VL, vicarious learning; PH, physiological and affective state; ACT, access to training; SUP, supervisor support of training; CSE, counseling self-efficacy. Number and text in bold indicate the non-linear relationship. * indicate the interaction effect of the exogenous/endogenous construct and the quadratic indicator.

In addition, we used the two stages approach in SmartPLS to measure the interaction term of the quadratic effect between each construct in the model, we found that only the relationship between social persuasion and counseling self-efficacy is subject to non-linearity. The result showed that increasing social persuasion has a positive but diminishing effect on counseling self-efficacy. However, the strength of this non-linear relationship is small as reported in its effect size (*f*^2^ = 0.007) (Kenny, 2018).

### Assessment of Endogeneity

According to Hair et al. (2019), researchers should only consider testing the endogeneity test when the focus of the research is on the model’s explanation and not testing the predictive causal orientation of the model. This is aligned with the present study where its’ main objective was on the predictive power of the variables namely mastery experience, social persuasion, vicarious learning, physiological and affective state, access to training, supervisor support on counseling self-efficacy, and job satisfaction. Additionally, we ran the Kolmogorov-Smirnov test using R studio (R Core Team, 2021) and found that the independent variables are all parametric, therefore we did not achieve the criteria to perform the Gaussian Copula to test the endogeneity of the model (Park and Gupta, 2012). As suggested by Hult et al. (2018), we reverted using the original PLS-SEM result.

### Assessment of Unobserved Heterogeneity

According to Sarstedt et al. (2017), the researchers could assess the unobserved heterogeneity of the PLS-SEM model using the finite mixture PLS (FIMIX-PLS) in SmartPLS. Thus, we conducted the FIMIX-PLS in 5 segments according to the Soper Test (Soper, 2021) with a minimum sample size of 100 assuming that the effect size is 0.15 and a power level of 0.8.

In Table 7, the results showed that AIC_3_ and CAIC point to a different segment, in which the former indicates the 5-segment while the latter indicates the 2-segment. Sarstedt et al. (2011) stated that AIC_4_ and BIC were the other indicators that served well to predict the number of segments in FIMIX-PLS. In our analysis, both of them point toward the 5-segment.

**TABLE 7:** Fit indices for the one- to five-segment solution.

	Number of segments				
Criteria	1	2	3	4	5
AIC	2681.61	2592.69	2542.71	2519.50	**2442.73**
AIC3	2690.61	2611.69	2571.71	2558.50	**2491.73**
AIC4	2699.61	2630.69	2600.71	2597.50	**2540.73**
BIC	2720.25	2674.27	2667.22	2686.95	**2653.11**
CAIC	2729.25	**2693.27**	2696.22	2725.95	2702.11
HQ	2696.72	2624.59	2591.40	2584.99	**2525.00**
MDL5	**2946.82**	3152.57	3397.26	3668.72	3886.62
LnL	–1331.81	–1277.35	–1242.36	–1220.75	–1172.36
EN	N/A	**0.60**	0.55	0.52	**0.60**
NFI	N/A	**0.69**	0.54	0.48	0.53
NEC	N/A	**215.12**	243.30	257.53	217.52

*AIC, Akaike’s Information Criterion; AIC3, Modified AIC with Factor 3; AIC4, Modified AIC with Factor 4; BIC, Bayesian Information Criteria; CAIC, Consistent AIC; HQ, Hannan Quinn Criterion; MDL5, Minimum Description Length with Factor 5; LnL, Log-Likelihood; EN, Entropy Statistic; NFI, Non-Fuzzy Index; NEC, Normalized Entropy Criterion; N/A, not available. Numbers in bold indicate the best outcome per segment retention criterion.*

Besides, the present study met the EN criterion, in which it was stated to be a minimum of 0.5 (Hair et al., 2016), while the single segment solution is preferred based on the relative segment sizes (Table 8). In addition, the analysis showed that MDL_5_ points toward the 1-segment, as suggested by Hair et al. (2016), the researchers should extract more segments as indicated by this criteria.

**TABLE 8:** Relative segment sizes (*N* = 500).

Number of segments	Segment 1	Segment 2	Segment 3	Segment 4	Segment 5
1	1.000				
2	0.900	0.100			
3	0.658	0.246	0.096		
4	0.490	0.242	0.227	0.042	
5	0.536	0.140	0.137	0.121	0.065

*The table shows the relative segment sizes in declining order per solution.*

In summary, the results did not point to a specific segmentation solution, precisely: (1) AIC_3_ and CAIC indicate different segments, and (2) AIC_4_ and BIC indicate a different segment than MDL_5_. Therefore, we concluded that there is no unobserved heterogeneity in our model.

## Discussion

This study examines the relationship of the sources of counseling self-efficacy, counseling self-efficacy, and job satisfaction, and, importantly, assesses the mediator role of counseling self-efficacy within the model.

### Mastery Experience, Social Persuasion, Access to Training, Supervisor Support of Training and Counseling Self-Efficacy

The very limited studies on Malaysian SCs’ self-efficacy warrants this study of the relationship of counseling self-efficacy sources, counseling self-efficacy, and job satisfaction for Malaysian SCs. The purpose is to enhance SCs’ contributions to students’ wellbeing. The rapidly changing environment as in, for example, COVID-19’s heavy impact on school education and student wellbeing underscores this.

The mastery experience source of self-efficacy yielded the *highest correlation* with counseling self-efficacy beliefs, consistent with previous studies that classified it as the most powerful information source to be retained and formed (Zelenak, 2015; Capa-Aydin et al., 2018). Mastery performance accomplishments contributed to the prediction of self-efficacy in general, as found in Capa-Aydin et al. (2018) and Ooi et al. (2018). The school counselors’ authentic experience of success in counseling cases (mastery) boosted their confidence and self-belief in delivering services and care. As counselors deal with various cases and vulnerable individuals, positive self-efficacy helps them believe they have the appropriate skillset and attributes, providing them with the confidence to tackle challenging counseling issues. Rather than addressing the task from a position of deficiency which affects levels of expected achievement, a positive sense of self-efficacy raises expectations of achievement, for the benefit of the clients. Thus, rather than with a sense of trepidation, counselors feel confident to respond to clients empathetically and perform excellently in counseling sessions.

The study finds that social persuasion such as feedback heightens CSE. Feedback reduced the discrepancy between counselors’ skill competency and perceived efficacy and cultivated better self-reflection and personal development. In particular, supervisor feedback increased the counselors’ confidence in their counseling competencies (Morrison and Lent, 2018). Thus, as part of the counselor-supervisor alliance, regular counseling supervision and on-time feedback sessions should be conducted to ensure ongoing evaluation of counseling skills and enhancement of counseling self-efficacy. The opportunity for feedback again raises the opportunity for counselors to discuss challenging counseling issues and, rather than ignoring areas where they need support, gain knowledge from supervisors of possible responses. In this way, the counselors’ issues are authenticated and perhaps normalized and seen as a normal part of counseling, supporting SCs’ personal development through reflection and deliberation.

In all, the study findings are consistent with previous research which indicated that access to training was positively associated with counselors’ self-efficacy in performing their duties and dealing with educational counseling, such as providing career guidance to students (Malkoç and Sünbül, 2020; Perry et al., 2020). The finding is aligned with Perry et al. (2020) study whereby counselors who received more training demonstrated a higher level of self-efficacy and better serve students and can assert one’s roles via effective communication in advocating the counselors’ and students’ roles and needs. It was also interesting that a study by Kozina et al. (2010) argued that training increased the overall measure of self-efficacy skills over 8 weeks, and micro-skills as a subdomain of CSE were found to significantly increase over time as compared to other subdomains. This further validates the idea that counselors’ self-efficacy could be increased, despite the period of training being short. Perhaps the access, instead of the duration, was the influencing factor.

It is also important that training opportunities must be associated with the SCs’ roles and functions, that is, contextualized. As part of employees’ compensation and benefits, training can be perceived as work environment support provided by the organization, again indicating to counselors that challenges and developing skills to address issues are not an individual deficiency, but the common path in school counseling. Thus, it is important to consider training as a multi-aspect, to promote job satisfaction for SCs. It is through training that counselors can face, express, and explore positively their concerns over challenging counseling situations and develop effective responses, raising their confidence and thus their levels of counseling self-efficacy. That is, training prepares counselors to deal positively with authentic experiences, explaining how the study’s findings are interwoven.

It is interesting to note that when assessed with environmental factors, supervisor support of training has a *higher correlation* with CSE than mastery experience. A positive supervisory working relationship increased the counselors’ willingness to disclose and perceive the evaluations and critiques received from supervisors as constructive feedback (Mehr et al., 2015). A collaborative supervisory alliance delivered higher clients’ satisfaction and produced positive therapeutic outcomes (Keum and Wang, 2021).

Additionally, the IPMA results indicated the supervisor support of training had the greater importance (i.e., being of high importance and performance) in shaping the CSE. Thus, in increasing SCs’ self-efficacy, schools with limited resources can priorities supervisors’ support for training. For changing circumstances such as the COVID-19 pandemic and with the increased use of technology in the delivery of counseling services, supervisors must allow school counselors to upskill in areas for which they have not been trained. The shift from meeting with clients face-to-face to the current remote meeting situation may lead to miscommunication in the therapeutic relationship. It may also hinder rapport building; thus, in online services, counselors may need to relearn how to build rapport, communicate and listen actively. Using online counseling services may also require SCs to update their ethical knowledge and practice to meet expected standards. That is, scheduling online appointments, selecting online platforms and video recording of sessions will require different protocols. All these would not be possible without the supervisors’ support for training opportunities.

### Counseling Self-Efficacy as a Mediator

Our study revealed that CSE only partially mediated the relationship between mastery experience, access to training, supervisor support toward training, and job satisfaction. Our finding asserted that mastery experience, access to training, and supervisor support of training predict CSE for Malaysian secondary SCs, which further contributes to their job satisfaction.

The study also contributes to the research gap in the application of SCT to self-efficacy and job satisfaction for SCs. As such, the importance of the bi-directional triadic reciprocal relationship among personal, environmental, and cognitive factors, as postulated by Bandura (1997) is corroborated. These findings point to SCs who reported a greater sense of job satisfaction having experienced, in combination, a higher degree of authentic experience, space and support for training, and a high level of CSE.

### Counseling Self-Efficacy and Job Satisfaction

Counseling self-efficacy was also reported as positively related to job satisfaction. This finding affirmed previous research where an individual with a higher level of self-efficacy demonstrates higher job satisfaction (Burić and Moe, 2020; Demir, 2020). To increase SCs’ job satisfaction, the cultivation of CSE among SCs is important. Counselor educators should emphasize SCs’ development of CSE beliefs through curriculum and continuous professional development programs. Additionally, registered counselors in Malaysia are required to demonstrate participation in continued education by obtaining 20 continuous professional development (CPD) points for their biyearly licensure and credentialing renewals.

The Board of Counselors Malaysia CPD strategy is divided into compulsory and elective categories. The CPD compulsory category focused on cultivating counseling competencies and any programs offered under this category must exhibit the element of “promoting and enhancing counseling competencies” as compared to the elective category which focused on general skill and competency development. Since counselors’ CSE are examined via three competencies, i.e., helping skills, counseling session management, and managing counseling challenges, the CPD programs shall focus on these and with such a strategy, SCs could better develop their counseling competencies and actions for a greater sense of job satisfaction.

### Limitations and Recommendations for Future Research

The methodology to collect the data was based solely on self-reported measures and the use of a single source method, which could lead to socially desired bias. To overcome this limitation, the actual measurement or a score may be collected via the yearly appraisal conducted by the school headmasters, or researchers could obtain data from multiple sources (e.g., clients, parents, supervisors, or colleagues). However, this process was not feasible in this study as the participants were approached randomly, and matching the appraisal records would violate ethical confidentiality and anonymity.

As per Bandura (1997), an individual’s physiological and affective state may not always be negative or linear. Usher (2009), using the qualitative method, found that students with high self-efficacy perceived physiological and affective states in the form of positive emotion and motivation, compared with students with low self-efficacy who perceived the physiological and affective state as stress and tension. Considering this, future investigation of this relationship could adopt the qualitative or mixed-method approach via interviews and the collection of data through personalized and individual reporting.

### Implications for School Counselors

This study’s key theoretical contribution is the conceptual framework design that improves the researcher’s knowledge of Malaysian SCs’ job satisfaction, with the main focus on the sources of counseling self-efficacy and CSE. The study further confirms the essential role of self-efficacy as a predictor of a greater positive work attitude and higher work satisfaction (Honicke and Broadbent, 2016). This study echoes Bandura’s Social Cognitive Theory (Bandura, 2012a) by incorporating personal and other cognitive, environmental, and behavioral determinants. Importantly, the study contributes in that it has extended and expanded the SCT concept and incorporated the organizational behavioral aspect through examining the six factors in relation to CSE and job satisfaction.

The major implication is that, firstly, this study contributes to the scenario above of well-balanced and effective SCs, and importantly given the impact of positive self-efficacy on work and job satisfaction, they can be used to identify SCs in need of assistance in cultivating their counseling self-efficacy. That is, drawing on Datu and Mateo (2016), counselors exhibiting CSE tended to believe they could perform their counseling tasks effectively and are more engaged in this. In the quest to promote counselors’ positive self-efficacy, our study provides guidance to the decision to assess, evaluate, identify, and predict SCs who are most likely to experience job satisfaction. Combined with the finding on the significance of training and supervisors’ support for it, the use of such an evaluation tool in targeting relevant counselors can lead to focussed content in training programs. School counselors who experienced lower counseling self-efficacy may require more guidance, assistance, or supervision to increase job satisfaction and deliver better therapeutic results.

The study paves the way for a theoretical understanding of counselors’ job satisfaction in the Malaysian context. Job satisfaction among educators, teachers, and other mental health professionals has commonly been studied, but there have been very few studies on secondary SCs. As put forward earlier, through their role in schools, counselors can impact vulnerable young people positively when they are at a formative stage in their lives and characters and so it is important that the counselors themselves have positive self-efficacy in their jobs to tackle counseling challenges with empathy. Indirectly, this provides positive life role models (mastery) for their clients who may be quite vulnerable. The study’s data is thus vital as it can be used to propose a healthy, balanced and comprehensive lifestyle model for SCs that considers the personal, environmental, cognitive, and behavioral determinants.

The last implication arises from the study’s finding that the cognitive self-efficacy aspect, although marginal, contributes further to counselors’ job satisfaction and as underpinning this discussion, the effectiveness of counselors in their roles. This finding more specifically indicates the four factors that contribute to counselors’ job satisfaction are: SCs’ own successful experience, counseling self-efficacy, access to training, and supervisor support of training. The study has also shown the priority of these factors as well as how they may be interwoven in increasing counselors’ self-efficacy and thus job satisfaction. Hence, this study’s data are informative for Malaysian professional counseling associations as they lead to a better and thorough understanding of SCs’ job satisfaction and thus interventions to support it. The school administrators, counseling supervisors, and counselor educators should play an active role in this process by providing platforms that encourage direct learning experience (mastery experience), promote, and cultivate the sense of counseling self-efficacy and ensure access and supportive supervisory relationship in training to the school counselors. These are the core issues addressed in this study.

## Conclusion

The purpose of this study was to determine the relationship between SCs’ counseling self-efficacy, and job satisfaction in Malaysia. The results of the PLS-SEM study identified four significant factors of job satisfaction: mastery experiences, access to training, perceived supervisor support of training, and counseling self-efficacy. Together, these factors predicted 13.2% of the variance in job satisfaction. Although only four factors are identified, the results support the proposed theoretical framework whereby efficacy information (i.e., mastery experience), environmental determinants (i.e., access to training and supervisor support of training), and cognitive determinant (i.e., counseling self-efficacy) corresponded congruently and lead to job satisfaction. Supervisor support of training is the strongest predictor of counseling self-efficacy.

## Data Availability Statement

The raw data supporting the conclusions of this article will be made available by the authors, without undue reservation.

## Ethics Statement

The studies involving human participants were reviewed and approved by the Ministry of Education, Malaysia-KB (BPPDP)603/5/JLD.07(20). The patients/participants provided their written informed consent to participate in this study.

## Author Contributions

PBO originated the design of the study, collected data, and performed the statistical analysis. PBO, WMWJ, and GC interpreted, drafted, and critically revised the draft manuscript. All authors have read and approved the final manuscript.

## Conflict of Interest

The authors declare that the research was conducted in the absence of any commercial or financial relationships that could be construed as a potential conflict of interest.

## Publisher’s Note

All claims expressed in this article are solely those of the authors and do not necessarily represent those of their affiliated organizations, or those of the publisher, the editors and the reviewers. Any product that may be evaluated in this article, or claim that may be made by its manufacturer, is not guaranteed or endorsed by the publisher.

## Footnotes

1
https://www.random.org/lists/

## References

[B1] Abdul Rahman A. M. Mohd Isa N. J. Atan A. (2013). A guidance and counseling model practiced within Malaysian schools. *Int. J. Educ. Res.* 1 1–12.

[B2] Akinlolu A. D. Chukwudi A. R. (2019). Counselling self-efficacy and professional commitment: the mediating role of emotional intelligence and gender identification. *Int. J. Sci. Res. Publ.* 9 2250–3153. 10.29322/IJSRP.9.03.2019.p8785

[B3] Al Haikal M. H. Saputra B. R. Adha M. A. Ariyanti N. S. (2020). “The efforts of young counselors to innovate counseling services in schools,” in *Proceedings of the 6th International Conference on Education and Technology (ICET 2020)*, (Dordrecht: Atlantis Press), 240–244.

[B4] Aliyev R. Tunc E. (2015). Self-efficacy in counseling: the role of organizational psychological capital, job satisfaction, and burnout. *Proc. Soc. Behav. Sci.* 190 97–105. 10.1016/j.sbspro.2015.04.922

[B5] Bagheri E. Jaafar W. M. B. W. Baba M. B. (2012). University climate and counseling students’ self-efficacy. *J. Educ. Soc. Res.* 2 95.

[B6] Bakioglu F. Türküm A. S. (2020). Investigation of the relationships among psychological counselor candidates’ counseling self-efficacy, multicultural competence, gender roles, and mindfulness. *Int. J. Prog. Educ.* 16 223–239. 10.29329/ijpe.2020.248.17

[B7] Bandura A. (1977). Self-efficacy: toward a unifying theory of behavioral change. *Psychol. Rev.* 84 191–215. 10.1037/0033-295X.84.2.191 847061

[B8] Bandura A. (1986). *Social Foundations of Thought and Action.* Englewood Cliffs, NJ: Prentice Hall, 23–28.

[B9] Bandura A. (1991). Social cognitive theory of self-regulation. *Organ. Behav. Hum. Decis. Process.* 50 248–287. 10.1016/0749-5978(91)90022-l

[B10] Bandura A. (1993). Perceived self-efficacy in cognitive development and functioning. *Educ. Psychol.* 28 117–148. 10.1207/s15326985ep2802_3

[B11] Bandura A. (1997). *Self-Efficacy: The Exercise of Control.* New York, NY: W H Freeman.

[B12] Bandura A. (2012b). On the functional properties of perceived self-efficacy revisited. *J. Manag.* 38 9–44. 10.1177/0149206311410606

[B13] Bandura A. (2012a). “Going global with social cognitive theory: from prospect to paydirt,” in *Applied Psychology*, eds Donaldson S. I. Berger D. E. Pezdek K. (New York, NY: Psychology Press), 65–92.

[B14] Bandura A. Walters R. H. (1977). *Social Learning Theory*, Vol. 1. Englewood Cliffs, NJ: Prentice-hall.

[B15] Britner S. L. Pajares F. (2006). Sources of science self-efficacy beliefs of middle school students. *J. Res. Sci. Teach.* 43 485–499. 10.1002/tea.20131

[B16] Bulut C. Culha O. (2010). The effects of organizational training on organizational commitment. *Int. J. Train. Dev.* 14 309–322.

[B17] Burić I. Moe A. (2020). What makes teachers enthusiastic: the interplay of positive affect, self-efficacy and job satisfaction. *Teach. Teach. Educ.* 89:103008. 10.1016/j.tate.2019.103008

[B18] Capa-Aydin Y. Uzuntiryaki-Kondakci E. Ceylandag R. (2018). The relationship between vicarious experience, social persuasion, physiological state, and chemistry self-efficacy: the role of mastery experience as a mediator. *Psychol. Sch.* 55 1224–1238. 10.1002/pits.22201

[B19] Chang Y. Edwards J. K. (2015). Examining the relationships among self-efficacy, coping, and job satisfaction using social career cognitive theory: an SEM analysis. *J. Career Assess.* 23 35–47. 10.1177/1069072714523083

[B20] Chin W. W. Marcolin B. L. Newsted P. R. (2003). A partial least squares latent variable modeling approach for measuring interaction effects: results from a Monte Carlo simulation study and an electronic-mail emotion/adoption study. *Inf. Syst. Res.* 14 189–217.

[B21] Cohen J. (1988). *Statistical Power Analysis for the Behavioral Sciences*, 2nd Edn. Hillsdale, NJ: Lawrence Erlbaum Associates.

[B22] Conner M. Norman P. (2015). *Predicting and Changing Health Behaviour: Research and Practice with Social Cognition Models*, 3rd Edn. Maidenhead: Open University Press.

[B23] Datu J. A. D. Mateo N. J. (2016). Perceived autonomy support moderates the relations between counseling self-efficacy and flow among Filipino counselors. *Curr. Psychol.* 35 69–76. 10.1007/s12144-015-9358-2

[B24] Demir S. (2020). The role of self-efficacy in job satisfaction, organizational commitment, motivation and job involvement. *Eurasian J. Educ. Res.* 20 205–224. 10.1046/j.1365-2834.1999.00129.x 10786546

[B25] Ellis D. S. (2019). *Exploring the Mediating Effects Between Counselor Self-Efficacy, Career Sustaining Behaviors, Perceived Wellness, and Burnout Among Novice Counselors: Testing Two Proposed Mediation Models*. Doctoral dissertation. Huntsville, TX: Sam Houston State University.

[B26] Geweke J. Meese R. (1981). Estimating regression models of finite but unknown order. *Int. Econ. Rev.* 22 55–70. 10.2307/2526135

[B27] Gold A. H. Malhotra A. Segars A. H. (2001). Knowledge management: an organizational capabilities perspective. *J. Manag. Inf. Syst.* 18 185–214. 10.1080/07421222.2001.11045669

[B28] Gundel E. Piro J. S. (2021). Perceptions of self-efficacy in mixed reality simulations. *Act. Teach. Educ.* 43 176–194. 10.1080/01626620.2020.1864513

[B29] Hair J. F. Black W. C. Babin B. J. Anderson R. E. (2010). *Multivariate Data Analysis: A Global Perspective*, 7th Edn. Upper Saddle River, NJ, USA: Pearson Education, Inc., 600–638.

[B30] Hair J. F. Hult G. T. M. Ringle C. M. Sarstedt M. (2017). *A Primer on Partial Least Squares Structural Equation Modeling (PLS-SEM)*, 2nd Edn. Los Angeles, CA: Sage Publication.

[B31] Hair J. F. Risher J. J. Sarstedt M. Ringle C. M. (2019). When to use and how to report the results of PLS-SEM. *Eur. Bus. Rev.* 31 2–24. 10.1108/EBR-11-2018-0203

[B32] Hair J. F. Sarstedt M. Hopkins L. Kuppelwieser V. G. (2014). Partial least squares structural equation modeling (PLS-SEM). *Eur. Bus. Rev.* 26 106–121.

[B33] Hair J. J. F. Sarstedt M. Matthews L. M. Ringle C. M. (2016). Identifying and treating unobserved heterogeneity with FIMIX-PLS: part I – method. *Eur. Bus. Rev.* 28 63–76. 10.1108/EBR-09-2015-0094

[B34] Harun M. M. (2015). Influence of Self-Perceived Multicultural Counselling Competence on Counselling Self-Efficacy Among Counselling Teachers Universiti Putra Malaysia. Available online at: http://psasir.upm.edu.my/id/eprint/59234/1/FPP%202015%207%20edited.pdf (accessed May 1, 2020).

[B35] Henseler J. Ringle C. M. Sarstedt M. (2015). A new criterion for assessing discriminant validity in variance-based structural equation modeling. *J. Acad. Mark. Sci.* 43 115–135. 10.1007/s11747-014-0403-8

[B36] Henseler J. Ringle C. M. Sinkovics R. R. (2009). “The use of partial least squares path modeling in international marketing,” in *New Challenges to International Marketing*, eds Sinkovics R. R. Ghauri P. N. (Bingley: Emerald Group Publishing Limited). 10.2196/jmir.3122

[B37] Honicke T. Broadbent J. (2016). The influence of academic self-efficacy on academic performance: a systematic review. *Educ. Res. Rev.* 17 63–84. 10.1016/j.edurev.2015.11.002

[B38] Hu B. Duan C. Jiang G. Yu L. (2015). The predictive role of mastery experience in Chinese counselors’ counseling self-efficacy. *Int. j. Adv. Couns.* 37 1–16. 10.1007/s10447-014-9221-4

[B39] Huang S. Yin H. Lv L. (2019). Job characteristics and teacher well-being: the mediation of teacher self-monitoring and teacher self-efficacy. *Educ. Psychol.* 39 313–331. 10.1080/01443410.2018.1543855

[B40] Hult G. T. M. Hair J. F. Proksch D. Sarstedt M. Pinkwart A. Ringle C. M. (2018). Addressing endogeneity in international maketing applications of partial least squares structural equation modeling. *J. Int. Mark.* 26 1–21. 10.1509/jim.17.0151 11670861

[B41] Iroegbu M. N. (2015). Self-efficacy and work performance: a theoretical framework of Albert Bandura’s model, review of findings, implications and directions for future research. *Psychol. Behav. Sci.* 4 170–173. 10.11648/j.pbs.20150404.15

[B42] Isaac O. Abdullah Z. Ramayah T. Mutahar A. M. (2017). Internet usage, user satisfaction, task-technology fit, and performance impact among public sector employees in Yemen. *Int. J. Inf. Learn. Technol.* 34 210–241. 10.1108/ijilt-11-2016-0051

[B43] Kenny D. A. (2018). *Moderation.* Available online at: http://davidakenny.net/cm/moderation.htm (accessed May 14, 2020).

[B44] Keum B. T. Wang L. (2021). Supervision and psychotherapy process and outcome: a meta-analytic review. *Transl. Issues Psychol. Sci.* 7 89–108. 10.1037/tps0000272

[B45] Kock N. Lynn G. S. (2012). Lateral collinearity and misleading results in variance-based sem: an illustration and recommendations. *J. Assoc. Inf. Syst.* 13 546–580. 10.17705/1jais.00302

[B46] Kounenou K. Gkemisi S. Nanopoulos P. Tsitsas G. (2018). The psychometric properties of the counselor burnout inventory in Greek school counsellors. *J. Psychol. Couns. Sch.* 28 33–54. 10.1017/jgc.2018.3

[B47] Kounenou K. Koumoundourou G. Makri-Botsari E. (2010). Greek school career counselors competencies and burnout syndrome. *Proc. Soc. Behav. Sci.* 2 1890–1895. 10.1016/j.sbspro.2010.03.1004

[B48] Kozina K. Grabovari N. Stefano J. D. Drapeau M. (2010). Measuring changes in counselor self-efficacy: further validation and implications for training and supervision. *Clin. Superv.* 29 117–127. 10.1111/jcpe.12368 25639948

[B49] Ku Johari K. S. Izwan M. Bali J. (2020). Psychological well being of school counselors. *Int. J. Psychosoc. Rehabil.* 24:2323.

[B50] Larson L. M. Daniels J. A. (1998). Review of the counseling self-efficacy literature. *Couns. Psychol.* 26 179–218. 10.1177/0011000098262001

[B51] Larson L. M. Suzuki L. A. Gillespie K. N. Potenza M. T. Bechtel M. A. Toulouse A. L. (1992). Development and validation of the counseling self-estimate inventory. *J. Couns. Psychol.* 39:105.

[B52] Lewandowski K. (2019). *Predictability of Supervisor Characteristics and Counselor Anxiety on Pre-Licensed Counselors’ Self-Efficacy*. Ph.D. thesis. Minneapolis, MN: Capella University.

[B53] Lin M. Wolke D. Schneider S. Margraf J. (2020). Bullying history and mental health in university students: the mediator roles of social support, personal resilience, and self-efficacy. *Front. Psychiatry* 10:960. 10.3389/fpsyt.2019.00960 31993000 PMC6971115

[B54] Low S. K. Kok J. K. Lee M. N. (2013). A holistic approach to school-based counselling and guidance services in Malaysia. *Sch. Psychol. Int.* 34 190–201. 10.1177/0143034312453398

[B55] Mahomed N. J. B. Johari K. S. K. Mahmud M. I. (2019). Coping strategies and psychological well-being of guidance and counselling teachers in schools. *Creat. Educ.* 10:3028. 10.4236/ce.2019.1012227

[B56] Malkoç A. Sünbül Z. A. (2020). The relationship between emotional literacy, cognitive flexibility and counseling self-efficacy of senior students in psychology and psychological counseling and guidance. *Educ. Res. Rev.* 15 27–33. 10.5897/ERR2019.3848

[B57] Mehr K. E. Ladany N. Caskie G. I. L. (2015). Factors influencing trainee willingness to disclose in supervision. *Train. Educ. Prof. Psychol.* 9 44–51. 10.1037/tep0000028

[B58] Morris D. B. Usher E. L. Chen J. A. (2017). Reconceptualizing the sources of teaching self-efficacy: a critical review of emerging literature. *Educ. Psychol. Rev.* 29 795–833.

[B59] Morrison M. A. Lent R. W. (2018). The working alliance, beliefs about the supervisor, and counseling self-efficacy: applying the relational efficacy model to counselor supervision. *J. Couns. Psychol.* 65 512–522. 10.1037/cou0000267 29999374

[B60] O’Neill S. Stephenson J. (2012). Exploring Australian pre-service teachers sense of efficacy, its sources, and some possible influences. *Teach. Teach. Educ.* 28 535–545. 10.1016/j.tate.2012.01.008

[B61] Ooi P.-B. Wan Jaafar W. M. Ang C.-S. Chan N.-N. (2020). Psychometric properties of the sources of counseling self efficacy in a sample of Malaysian secondary school counselors. *SAGE Open* 10:2158244020902076. 10.1177/2158244020902076

[B62] Ooi P. B. Wan Jaafar W. M. B. Baba M. B. (2018). Relationship between sources of counseling self-efficacy and counseling self-efficacy among Malaysian school counselors. *Soc. Sci. J.* 55 376–389.

[B63] Park S. Gupta S. (2012). Handling endogenous regressors by joint estimation using copulas. *Mark. Sci.* 31 567–586. 10.1287/mksc.1120.0718 19642375

[B64] Perry J. Parikh S. Vazquez M. Saunders R. Bolin S. Dameron M. L. (2020). School counselor self-efficacy in advocating for self: how prepared are we? *J. Couns. Prep. Supervis.* 13:5.

[B65] Peura P. Aro T. Räikkönen E. Viholainen H. Koponen T. Usher E. L. (2021). Trajectories of change in reading self-efficacy: a longitudinal analysis of self-efficacy and its sources. *Contemp. Educ. Psychol.* 64:101947. 10.1016/j.cedpsych.2021.101947

[B66] Phan H. P. Ngu B. H. (2016). Sources of self-efficacy in academic contexts: a longitudinal perspective. *Sch. Psychol. Q.* 31:548. 10.1037/spq0000151 26985966

[B67] R Core Team (2021). *R: A Language and Environment for Statistical Computing.* Vienna: R Foundation for Statistical Computing.

[B68] Ramayah T. Cheah J. Chuah F. Ting H. Memon M. (2018). *Partial Least Squares Structural Equation Modeling (PLS-SEM) Using smartPLS 3.0. An Updated Guide and Practical Guide to Statistical Analysis.* Kuala Lumpur: Pearson.

[B69] Ramsey J. B. (1969). Tests for Specification errors in classical linear least-squares regression analysis. *J. R. Stat. Soc. Ser. B Methodol.* 31 350–371. 10.1111/j.2517-6161.1969.tb00796.x

[B70] Rojo A. Llorens-Montes J. Perez-Arostegui M. N. (2016). The impact of ambidexterity on supply chain flexibility fit. *Supply Chain Manag. Int. J.* 21 433–452. 10.1108/SCM-08-2015-0328

[B71] Rojo A. Perez-Arostegui M. N. Montes F. J. L. (2020). Ambidexterity and IT competence can improve supply chain flexibility? A resource orchestration approach. *J. Purch. Supply Manag.* 26:100610. 10.1016/j.pursup.2020.100610

[B72] Rojo A. Stevenson M. Lloréns Montes F. J. Perez-Arostegui M. N. (2018). Supply chain flexibility in dynamic environments. *Int. J. Operat. Prod. Manag.* 38 636–666. 10.1108/IJOPM-08-2016-0450

[B73] Sarstedt M. Becker J.-M. Ringle C. M. Schwaiger M. (2011). Uncovering and treating unobserved heterogeneity with FIMIX-PLS: which model selection criterion provides an appropriate number of segments? *Schmalenbach Bus. Rev.* 63 34–62. 10.1007/BF03396886

[B74] Sarstedt M. Ringle C. M. Cheah J.-H. Ting H. Moisescu O. I. Radomir L. (2020). Structural model robustness checks in PLS-SEM. *Tour. Econ.* 26 531–554. 10.1177/1354816618823921

[B75] Sarstedt M. Ringle C. M. Hair J. F. (2017). “Treating unobserved heterogeneity in PLS-SEM: a multi-method approach,” in *Partial Least Squares Path Modeling: Basic Concepts, Methodological Issues and Applications*, eds Latan H. Noonan R. (Cham: Springer International Publishing), 197–217. 10.1007/978-3-319-64069-3_9

[B76] Schiele B. E. (2013). *The Importance of Counselling Self-Efficacy in School Mental Health*. Master’s thesis. Columbia, SC: University of South Carolina.

[B77] Schunk D. H. (2017). “Motivation and social cognitive theory,” in *Proceedings of the Motivation Theory Yesterday, Today, and Tomorrow: Reflections of founders and descendants (Symposium)*, eds Koenka A. C. Wigfield A. L. (San Antonio, TX: American Educational Research Association).

[B78] Schunk D. H. DiBenedetto M. K. (2020). Motivation and social cognitive theory. *Contemp. Educ. Psychol.* 60:101832. 10.1016/j.cedpsych.2019.101832

[B79] Schwarz G. (1978). Estimating the dimension of a model. *Ann. Stat.* 6 461–464. 10.1007/978-3-319-10470-6_18

[B80] Sharma P. N. Shmueli G. Sarstedt M. Danks N. Ray S. (2021). Prediction-oriented model selection in partial least squares path modeling. *Decis. Sci.* 52 567–607. 10.1111/deci.12329

[B81] Shmueli G. Ray S. Estrada J. M. V. Chatla S. B. (2016). The elephant in the room: predictive performance of PLS models. *J. Bus. Res.* 69 4552–4564. 10.1016/j.jbusres.2016.03.049

[B82] Shmueli G. Sarstedt M. Hair J. F. Cheah J.-H. Ting H. Vaithilingam S. (2019). Predictive model assessment in PLS-SEM: guidelines for using PLSpredict. *Eur. J. Mark.* 53 2322–2347. 10.1108/EJM-02-2019-0189

[B83] Soper D. S. (2021). A-Priori Sample Size Calculator for Structural Equation Models [Software]. Available online at: https://www.danielsoper.com/statcalc

[B84] Svensson G. Ferro C. Høgevold N. Padin C. Varela J. C. S. Sarstedt M. (2018). Framing the triple bottom line approach: direct and mediation effects between economic, social and environmental elements. *J. Clean. Prod.* 197 972–991. 10.1016/j.jclepro.2018.06.226

[B85] Talsma K. Schüz B. Schwarzer R. Norris K. (2018). I believe, therefore I achieve (and vice versa): a meta-analytic cross-lagged panel analysis of self-efficacy and academic performance. *Learn. Individ. Dif.* 61 136–150.

[B86] Tan V. (2021). *Parents in Malaysia Fret Over Academic Progress Amid Prolonged School Closure, Online Learning.* Queenstown: Channel News Asia.

[B87] Tehseen S. Ramayah T. Sajilan S. (2017). Testing and controlling for common method variance: a review of available methods. *J. Manag. Sci.* 4 142–168. 10.20547/jms.2014.1704202

[B88] Ümmet D. (2017). Structural relationships among counselling self-efficacy, general self-efficacy and positive-negative affect in psychological counsellor candidates. *Educ. Sci. Theory Pract.* 17 1875–1892. 10.12738/estp.2017.6.0180

[B89] Usher E. L. (2009). Sources of middle school students’ self-efficacy in mathematics: a qualitative investigation. *Am. Educ. Res. J.* 46 275–314.

[B90] Usher E. L. Pajares F. (2009). Sources of self-efficacy in mathematics: a validation study. *Contemp. Educ. Psychol.* 34 89–101.

[B91] Watson J. C. (2012). Online learning and the development of counseling self-efficacy beliefs. *Prof. Couns.* 2 143–151.

[B92] Weiss D. J. Dawis R. V. England G. W. (1967). *Manual for the Minnesota Satisfaction Questionnaire. Minnesota Studies in Vocational Rehabilitation.* Minneapolis: University of Minnesota.

[B93] Wilson C. Marks Woolfson L. Durkin K. (2020). School environment and mastery experience as predictors of teachers’ self-efficacy beliefs towards inclusive teaching. *Int. J. Inclusive Educ.* 24 218–234. 10.1080/13603116.2018.1455901

[B94] Yusoff M. S. B. Hadie S. N. H. Mohamad I. Draman N. Al-Aarifin I. M. Rahman W. F. W. A. (2020). Sustainable medical teaching and learning during the COVID-19 pandemic: surviving the new normal. *Malays. J. Med. Sci.* 27 137. 10.21315/mjms2020.27.3.14 32684814 PMC7337950

[B95] Zelenak M. S. (2015). Measuring the sources of self-efficacy among secondary school music students. *J. Res. Music Educ.* 62 389–404.

